# Increased adherence to influenza vaccination among Palermo family pediatricians: a study on safety and compliance of qLAIV vaccination

**DOI:** 10.1186/s13052-024-01693-y

**Published:** 2024-07-11

**Authors:** Claudio Costantino, Fabio Tramuto, Nicole Bonaccorso, Maria Carmela Lo Giudice, Francesco Balsamo, Alessandro Carubia, Luciano D’Azzo, Santo Fruscione, Martina Sciortino, Tania Vitello, Luigi Zagra, Alessia Pieri, Rosaria Rizzari, Gregorio Serra, Mario Palermo, Maria Angela Randazzo, Sara Palmeri, Rosario Asciutto, Giovanni Corsello, Giorgio Graziano, Carmelo Massimo Maida, Walter Mazzucco, Francesco Vitale

**Affiliations:** 1 Department of Health Promotion, Maternal and Infant Care, Internal Medicine and Excellence Specialties “G. D’Alessandro”, University of Palermo, Palermo, Italy; 2 Hospital Unit of Clinical Epidemiology with Cancer Registry, University Hospital “Paolo Giaccone” of Palermo, Palermo, Italy; 3 Italian Federation on Family Paediatricians, Sicilian Section, Palermo, Italy; 4 Regional Health Authority of Sicily, Palermo, Italy; 5 Prevention and Epidemiology Unit, Palermo Local Health Authority, Palermo, Italy; 6 PhD National Programme in One Health approaches to infectious diseases and life science research, Departiment of Public Health, Experimental and Forensice Medicine, University of Pavia, Pavia, 27100 Italy

**Keywords:** Influenza, Vaccination, qLAIV, Safety, Pediatrics

## Abstract

**Background:**

Influenza represents a serious public health threat, especially for the management of severe cases and complications of the disease, requiring the implementation of control measures. We aimed to assess the acceptance and impact of qLAIV vaccination among a representative sample of family paediatricians (FPs) operating in Palermo Local Health Authority (LHA). To this end we evaluated vaccination coverage rates, comparing it with that observed in Sicilian context, while actively monitoring possible adverse reactions and their severity.

**Methods:**

An observational descriptive non-controlled study was conducted in two phases, from September 2022 to June 2023. The first phase involved a formative and educational intervention with a pre-intervention questionnaire to assess the knowledge and attitudes of FPs on paediatric influenza vaccination. The second phase consisted of an active surveillance on qLAIV safety and acceptance among the paediatric population assisted by the participating FPs, from October 2022 to April 2023. Frequencies, chi-squared tests, and comparisons statistics were performed using Stata/MP 14.1.

**Results:**

The overall coverage rate among the paediatric population involved in the intervention was 13.2%, with an I.M./qLAIV ratio of vaccine administered of 1/4.25. This coverage rate was significantly higher (*p*-value <0.001) when compared to the average values reported in the population under the Palermo Local Health Authority (LHA) (6.7%) and in the entire Sicily (5.9%). Adverse events in the qLAIV group were mild, with only 3.3% experiencing them, primarily presenting as a feverish rise (3.2%). No severe adverse reaction was reported.

**Conclusions:**

The educational intervention significantly raised paediatric influenza vaccination rates among the participating FPs, and in general improved influenza vaccination coverage rates in the Palermo’s LHU. Minimal, non-serious adverse events underscored the vaccine’s safety. Training sessions ensured paediatricians stayed informed, enabling them to provide comprehensive information to parents for secure and informed vaccination decisions in their practices.

## Introduction

Influenza represents a serious public health threat, especially for the management of severe cases and complications of the disease, therefore requiring the implementation of control measures. It also represents one of the few infectious diseases that every individual experiences several times during their existence regardless of lifestyle, age, and where they live. The most vulnerable population categories such as young children, the elderly and patients with comorbidities, are at greater risk of developing serious complications, including bacterial and viral pneumonia, and exacerbation of underlying medical conditions, which in some cases lead to hospitalization and death [1].

From an epidemiological point of view, it is estimated that worldwide influenza causes one billion cases and between three and five million cases of serious illness every year, while deaths amount to between 290,000 and 650,000 [2]. In Europe, according to the European Center for Disease Control (ECDC), between 4 and 50 million symptomatic cases of influenza occur, with 15,000/70,000 estimated deaths from flu-related causes occurring every year [3].

In Italy, influenza and its associated pneumonia are among the top 10 leading causes of death, and through the “Influnet” network it is possible to obtain useful information on the epidemiological trend of flu-like syndromes and on the virological surveillance of influenza [1].

Every year, in Italy, the incidence rate of influenza, although varying in the various seasons, is on average around 9 (range: 4–15) per 1,000 inhabitants in the general population, while in the 0–14 age group, the most affected one, incidence averages about 26 per 1,000 inhabitants (range: 12–40) [4].

However, the data may be underestimated because the flu symptoms can be confused with those of other viral diseases which lead the patient not to seek medical attention [1].

The goal of the vaccination program is to provide direct protection to all children, as they are the main spreaders of the flu and are a crucial reservoir of the virus [5]. At the same time, vaccination of paediatric population provides indirect protection against the adult population [6].

Live attenuated influenza vaccine has been approved for use in many States in Europe [7] but also in the United States [8] and Canada [9]. Live attenuated influenza vaccine, authorized by the European Medicines Agency (EMA) between 2 and 17 years, is a non-invasive intranasal quadrivalent live attenuated vaccination (qLAIV). The ability to induce immune responses at the site of infection, eliciting IgA response, represents one of the main advantages [10], while a recent comparative study between qLAIV and inactivated quadrivalent influenza vaccine standard dose (QIVsd) showed that the intranasal vaccine is much more effective in preventing influenza severe cases [11, 12].

In Italy, influenza vaccination coverage among paediatric population remains generally low [13].

The report of the Italian Ministry of Health for the 2022–2023 season documented a 7.2% coverage in the age group between 6 and 23 months, 9.2% in the 2–4 years old cohort, 26.6% between 5 and 8 years old children, 4.9% between 9 and 14 years old, and 2.1% between 15 and 17 years old [13].

In light of these evidence, we conducted an observational study to explore the acceptance rate and the impact of the qLAIV vaccination into the paediatric vaccination offer in the Palermo Local Health Authority (LHA) corresponding to the Palermo metropolitan area, the most populous area in Sicily (the fourth Region by population in Italy) with more than 1.2 billion inhabitants and an average of 10,000 yearly births over the last decade [14].

More in depth, we evaluated vaccination coverage rates within the paediatric population assisted by a sample of the family paediatricians (FPs) involved in a formative intervention, comparing it with adherence rates observed in the Palermo’LHA and Sicilian Region. Moreover, an active surveillance of adverse reactions related to the qLAIV vaccination, and their severity was carried out.

This comprehensive approach aimed to shape strategies that would bolster vaccination coverage, thereby mitigating the risk of vaccine-preventable infectious diseases.

## Methods

An observational descriptive study, including a non-controlled educational intervention, was conducted to evaluate the safety of the intranasal spray influenza vaccine (qLAIV) and the vaccine adherence among a sample of FPs operating in the Palermo LHA. The study was designed within a two phases project coordinated by the Hygiene Section of the Department of Sciences for Health Promotion, Maternal and Infant Care, Internal Medicine, and Excellence Specialties “G. D’Alessandro” of the University of Palermo, in collaboration with the Palermo LHA’s Health Department and the Provincial Section of the Italian Federation of Family Paediatricians.

### Phase 1: introduction of qLAIV vaccination and formative intervention

The target of the formative intervention was represented by the 62 FPs practicing within the Palermo Local Health Authority (LHA).

The qLAIV vaccination, encompassing effectiveness, administration method, and safety aspects, was introduced during a continuing medical education event, hold on September 23rd, 2022, attended by 41 FPs out of 62 (Response Rate: 66.12%), recruited by the Palermo Section of the Italian Federation of Family Paediatricians (FPs). Before the formative intervention, participants received an anonymous questionnaire to be self-administered via a QR code on the Google^®^ Module platform. The questionnaire aimed to previously explore willingness, acceptance, attitudes, and knowledge regarding paediatric influenza vaccination.

Following the formative session, information on the project’s rationale, qLAIV characteristics, other influenza vaccines available for the paediatric population, administration methods, organizational strategies, and the contents of the Sicilian regional recommendations for the seasonal influenza vaccination campaign 2022/2023, was provided.

### Phase 2: active surveillance on qLAIV safety and vaccination adherence

Throughout the year, two brief online webinars were organized on January 11, 2023, and April 5, 2023, presenting ongoing epidemic and virological influenza trend during 2022/2023 season, including vaccinations administered in the Palermo LHA, preliminary data on active surveillance on adverse events reported by the participating FPs. Discussions also included data on responses to the initial questionnaire administered to FPs at the first event.

The project finished on June 16, 2023, with the presentation of data on the adherence to the seasonal vaccination campaign among the paediatric population in the study, as compared with the paediatric population of the Palermo LHA and Sicily, along with safety data collected via the surveillance system.

#### Active surveillance on qLAIV safety among vaccinated individuals and adherence to influenza vaccination in the paediatric population

In accordance with the Directive 2001/20/EC Art. 2, adverse events were defined as:

- Expected adverse event (EAEs): expected reaction listed in the data sheet.
- Unexpected adverse event (UAEs):

1. serious unexpected adverse avent: a serious adverse reaction whose nature, severity, or outcome is not consistent with the reference safety information.
2. serious adverse event: any harmful clinical event that, regardless of the dose, requires hospitalization or prolongs ongoing hospitalization, results in severe or prolonged disability or incapacity, results in a congenital anomaly or birth defect, is life-threatening, or causes death [15].

Parents actively participated in surveillance by completing a diary form, either at the time of the second dose or reporting via telephone to the paediatrician starting on the 10th day post-vaccination. The diary card, designed with detailed instructions for proper completion and organized into sections, functioned as a comprehensive tool.

It provided guidance on reporting expected adverse events, ensuring that parents were well-informed about events listed in the data sheet, thus facilitating prompt communication with their paediatrician.

Utilizing the same card, parents monitored specific adverse events based on the summary of product characteristics (SmPC) through eight questions.

This included investigating skin rash, headache, general malaise, muscle pain, and inappetence, specifying the intensity and duration of the symptoms. It also covered monitoring temperature, specifying degree and duration, and hypersensitivity reactions, specifying facial oedema, urticaria, other severe anaphylactic reaction, with respiratory or circulatory involvement.

All expected adverse events were to be indicated as absent, mild (mild symptoms that typically resolve within 24 h without the need for medications), moderate (moderate-intensity symptoms that generally resolve within 24 h with the administration of appropriate medications), or severe (severe symptoms that are typically resolved with the administration of medications within 72 h).

Simultaneously, they shared their perceptions of qLAIV compared to intramuscular vaccination, if previously administered, and offered insights for potential enhancements in future paediatric flu campaigns.

#### Indication and recommendation of qLAIV vaccine in accordance with SmPC and recommendations of Sicilian region for 2022/23 season

Children without contraindications to qLAIV vaccine, excluding patients under 24 months of age, allergic to egg proteins or gentamicin, immunosuppressed, or with severe asthma, were considered for the study. Ethical approval was obtained from the University Hospital of Palermo Ethical Committee (session of July 2022, protocol no. 07/2022).

### Statistical analysis

The data obtained were uploaded in a Microsoft Excel^®^ spreadsheet (which were also automatically generated by the Google ^®^ Modules online questionnaire collection system by the FPs).

Data were distributed and summarized as means with their standard deviations. Absolute and relative frequencies were calculated for qualitative variables. Chi-squared tests (with Fisher’s correction, where appropriate) were used to compare categorical variables and especially to compare the significance of the increase in vaccination coverage rates between Sicily, Palermo, and the intervention group.

All the data were analysed using the statistical software package Stata/MP 14.1 (StataCorp LP, College Station, TX, USA).

## Results

The total number of participants to the baseline survey for the pre- intervention training questionnaire was of 26 FPs out of the 41 participants (RR:63.41%). As reported in Table 1, respondents were predominantly female (84.6%). Most respondents (61.5%) correctly identified the administration ways of paediatric influenza vaccines available in Italy, such as intramuscular and/or intranasal. Only 26.9% correctly identified the authorized type of influenza vaccine for children aged 6 to 24 months (e.g., standard dose quadrivalent inactivated - QIVsd).

Further, 46.2% correctly identified the authorized type of vaccine for children older than 25 months (cell-cultured quadrivalent inactivated - QIVcc), while 76.9% correctly identified the live attenuated quadrivalent intranasal - qLAIV. Correct responses for other vaccine types were lower. Regarding regional recommendations for children under 9 y.o. of age vaccinated for the first time, 84.6% of respondents provided correct answers, emphasizing the need for a second booster dose 4 weeks apart. Among participants, 46.2% accurately identified contraindications for qLAIV vaccine administration in children (e.g., immunocompromised, and allergic to egg), while 65.4% provided correct answers regarding contraindications for the administration of inactivated influenza vaccines.

In addition, 76.9% indicated medical interviews with parents/family members/legal guardians as a tool to evaluate patients’ vaccination history, and 50% of the sample positively evaluated the communicative strategies of the Palermo Local Health Authority on paediatric influenza vaccination. In terms of knowledge about the definitions of upper respiratory tract infections (ILIs) and lower respiratory tract infections (ARIs) by the European Centre for Disease Prevention and Control (ECDC), 50.0% of participants provided correct answers.

Additionally, only 23.1% of participants accurately identified the duration of the Respivirnet epidemiological and virological surveillance system, responding October-epidemiological/November-virological to April for both.

**Table 1:** Willingness of administration, acceptance, attitudes, and knowledge on paediatric influenza vaccination self-reported by the family paediatricians before the formative intervention (*n* = 26 respondents on 41 participants)

Question	*n*. (%)
Gender	
*- female*	22 (84.6%)
*- male*	4 (15.4%)
Knowledge on administration ways of paediatric influenza vaccines available in Italy	
*- correct answer (e.g. intramuscular and/or intranasal)*	16 (61.5%)
*- incorrect answer (e.g. subcutaneous administration)*	10 (38.5%)
Type of influenza vaccine authorized from 6 to 24 months of age	
*- correct answer (e.g. standard dose quadrivalent inactivated - QIVsd)*	7 (26.9%)
*- incorrect answer*	19 (73.1%)
Type of influenza vaccine authorized from 25 months of age	
*- correct answer (cell cultured quadrivalent inactivated - QIVcc)*	12 (46.2%)
*- correct answer (standard dose quadrivalent inactivated - QIVsd)*	7 (26.9%)
*- correct answer (live attenuated quadrivalent intrasasal - qLAIV)*	20 (76.9%)
*- incorrect answer (high dose quadrivalent inactivated - QIVhd)*	1 (3.8%)
*- incorrect answer (MF59- adjuvanted quadrivalent inactivated - QIVa)*	7 (26.9%)
Regional recommendations for children under 9 y.o. vaccinated for the first time	
*- correct answer (need of second booster dose 4 weeks apart)*	22 (84.6%)
*- incorrect answer*	4 (15.4%)
Contraindications of qLAIV vaccine administration among children	
*- correct answer (e.g. immunocompromised and allergic to egg)*	12 (46.2%)
*- incorrect answer*	14 (53.8%)
Contraindications of an inactivated influenza vaccine (QIVcc/QIVsd) administration among children	
*- correct answer*	17 (65.4%)
*- incorrect answer (e.g. immunocompromised or oncological)*	9 (34.6%)
Tools to evaluate vaccination history of the patients	
*- Regional vaccination registry/FPs personal medical registry*	6 (23.1%)
*- Medical interview with parents/family members/legal guardian;*	20 (76.9%)
Palermo LHA’s Communicative strategies on pediatric influenza vaccination	
*- excellent/good*	13 (50.0%)
*- acceptable/inadequate*	13 (50.0%)
Frequency of previous participation to formative events on vaccines	
*- more than one/at least once yearly*	21 (80.7%)
*- one in the last two/three years*	5 (19.3%)
Knowledge about ILIs/ARIs definitions of the ECDC	
*- correct answer (e.g. accordance to ECDC definition)*	13 (50.0%)
*- incorrect answer*	13 (50.0%)
Duration of the Respivirnet/Influnet epidemiological/virological surveillance system	
*- correct answer (October-epidemiological/November-virological – April)*	6 (23.1%)
*- incorrect answer*	20 (76.9%)

### Active surveillance on qLAIV safety and vaccine adherence

Among the 41 FPs involved in the research project, the vaccinable population comprised 4,850 children aged between 2 and 14 years. Of these, 1,939 (40%) were in the age class 2–6 years.

**Fig. 1:**
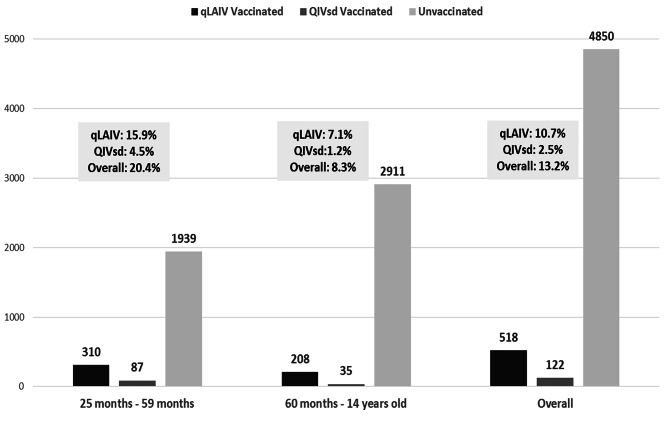
Adherence to paediatric influenza vaccination with qLAIV and QIVsd vaccine among the FPs involved in the study

Although 41 FPs were engaged in the formative intervention, only 17 (40%) actively participated by administering vaccinations and reporting vaccination data. Overall, 518 (10.7%) children received the qLAIV from FPs. This group comprised 310 (59.9%) children aged between 24 months and 6 years old, and 208 (40.1%) between 7 and 14 years. Notably, 1,939 patients (40%) were in the age group 2–6 years old (Fig. 1). Examining the two age groups separately (Figs. 1), 15.9% of the children in the first group (2–6 years old) received the qLAIV, compared to 7.1% the 7–14 years old group. Lastly, 122 children (2.5%) from both groups were vaccinated with the intramuscular vaccine.

**Fig. 2:**
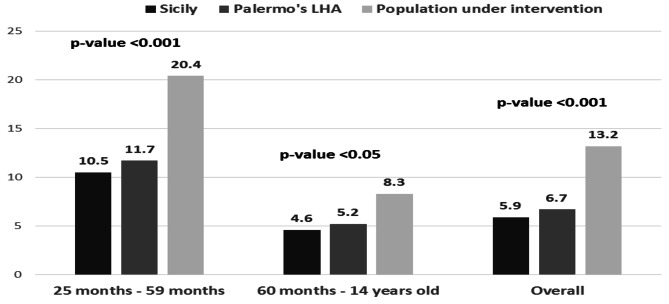
Vaccination coverage rates against influenza observed during 2022/23 season among the paediatric population: comparison between Sicilian region, Palermo LHA and intervention group

An analysis of influenza vaccination coverage rates during the 2022/23 season among the paediatric population in the Sicilian Region, the Palermo LHA, and the intervention population showed a higher vaccination coverage in the intervention population, as depicted in Fig. 2.

The values among the paediatric population of intervention showed an overall coverage rate of 13.2%, with an I.M./qLAIV ratio of 1/4.25, significantly higher (*p*-value < 0.001) than average values reported in the Palermo LHA (6.7%) and in Sicily (5.9%) (Fig. 2).

Furthermore, in the age class 25–59 months the coverage reached in the population of intervention (20.4%) was also significantly higher (*p*-value < 0.001) than the ones of Palermo LHA (11.7%) and Sicily (10.5%). Finally, also a significant increase of influenza vaccination coverage rate was observed among children aged 60 months-14 years old (8.3% in the population with intervention vs. 5.2% in the Palermo’s LHA and 4.6% in the Sicilian region).

Among those undergoing intranasal vaccination, only 17 (3.3%) experienced expected adverse events, all of which were mild and reported by paediatricians. The majority (3.2%) showed a feverish rise, with an average evening temperature of 38 °C degrees. Other reported symptoms included headache (1.3%), general malaise (0.09%), myalgia (0.03%), and nasal congestion (0.03%). No cases of hypersensitivity reactions, inappetence, or skin reactions were reported (Table 2).

**Table 2:** Expected and unexpected adverse events in the 7 days after the qLAIV vaccine doses administered. Data collected and monitored by a phone active surveillance system

qLAIV expected adverse events	*n*.	% on overall vaccine doses administered
- Fever > 38.5 °C (for more than 12 h)	10	3.2%
- Headache		
mild	4	1.3%
moderate	0	-
severe	0	-
- General malaise		
mild	3	0.09%
moderate	0	-
severe	0	-
- Myalgia		
Mild	1	0.03%
moderate	0	-
Severe	0	-
-Respiratory reactions		
Runny nose	1	0.03%
Nasal congestion	1	0.03%
Epistaxis	0	-
- Skin reactions	0	-
- Inappetence	0	-
qLAIV unexpected adverse events		
- Anaphylaxis	0	-
- Glottal edema	0	-
- Other severe respiratory symptoms	0	-

## Discussion

This study aimed to assess the acceptance of qLAIV vaccination and its impact on paediatric vaccination within Palermo Local Health Unit. Data on possible associated adverse reactions were also provided through an active surveillance.

The baseline survey conducted in September 2022 unveiled crucial insights into FPs’ knowledge and attitudes regarding paediatric influenza vaccination. Although many respondents accurately identified vaccination administration methods, a notable portion lacked knowledge about the specific vaccine types authorized for various age groups. Recognizing these gaps, it was essential to understand participants’ baseline perspectives before the intervention. This facilitated a thorough assessment of the subsequent training’s impact on their attitudes and knowledge, laying the groundwork for precise enhancements in vaccination practices and ultimately aiming to bolster vaccination coverage rates [16].

Previous research has highlighted the effectiveness of educational interventions aimed at pediatricians in promoting vaccination [17, 18]. These interventions often involve various strategies such as training sessions, webinars, and informational materials tailored to address specific vaccination-related concerns [17, 18]. By equipping pediatricians with up-to-date information and addressing any misconceptions or uncertainties they may have, these interventions have been shown to positively influence pediatricians’ attitudes towards vaccination and enhance their confidence in recommending vaccines to parents [19, 20]. As a result, they play a vital role in improving vaccination coverage rates among children, ultimately contributing to public health efforts to prevent vaccine-preventable diseases.

During the active surveillance phase, conducted from January to June 2023, a notable percentage of the involved FPs actively participated in administering influenza vaccines, indicating positive results in term of engagement towards promoting paediatric vaccination.

However, despite this overall interest, some paediatricians opted to address patients to nearby vaccination centers, instead of administering vaccines themselves. This limited involvement of FPs, accounting for only 40% of the total, posed challenges to achieving higher vaccination coverage. These challenges were primarily attributable to logistical issues, such as vaccine supply constraints and patient overload [21].

The two online webinars organized during this period provided participants with a platform to exchange information and experiences, presenting current results including the number of administered vaccinations, surveillance of adverse event data, and vaccine adherence among the paediatric population in Sicily. Notably, these webinars facilitated ongoing communication with participating paediatricians, contributing significantly to overall success of the study [22–24]. The safety of the qLAIV was assessed through an active surveillance system, with adverse events (AEs) documented at one-week intervals following qLAIV administration by paediatricians previously trained for this purpose. Adverse events were reported in a minority of patients and were predominantly mild in nature, characterized by symptoms such as fever, occasional headaches, and general malaise. Importantly, no participants experienced hypersensitivity reactions, loss of appetite, or skin-related issues. These findings highlight the overall safety of qLAIV, with infrequent adverse events primarily presenting as mild symptoms. This corroborates findings from a review previously published, although nasal congestion/runny nose emerged as the most common adverse event [25].

Our results suggest that the training provided to paediatricians equipped them with additional tools to communicate heightened awareness of qLAIV vaccine safety to parents.

Previous studies highlighted that childhood influenza vaccination significantly reduced the risk of paediatric intensive care unit admissions by 74%, emphasizing the importance of vaccination in mitigating severe influenza manifestations [26, 27].

Although children with severe asthma episodes were excluded based on intranasal spray vaccine recommendations, other evidence suggest that the intranasal vaccine may be tolerated in children with moderate to severe asthma episodes [28].

Examining the Palermo LHA, data indicated a higher utilization of the intranasal vaccine in the 2–6 age group compared to the 7–14 age group (60% vs. 40% of total qLAIV administered), that resulted also higher in our group of intervention (1:4.25). This is in partial contrast with another study, where parental preferences favoured qLAIV, only among children aged 6 years or older [29]. In comparison with Sicilian influenza vaccination adherence rates, the Palermo LHA demonstrated higher coverages for both age groups, despite not achieving notable increase (personal data of the Palermo’s LHA available on motivated request to the AAs). This outcome underscored the effectiveness of educational interventions conducted among paediatricians. Intriguingly, despite low overall vaccination coverage, intranasal vaccination emerged as the preferred choice in both age groups (2–6 years and 6–14 years). This preference may be attributed to the administration method of qLAIV, which is administered as a nasal spray. This characteristic is particularly important for promoting compliance among the paediatric population, as it offers a less invasive and more easily tolerated option as compared to other influenza vaccines administered via injection.

Our results suggested that standardizing formative intervention for healthcare professionals (HCPs) on vaccination offer is of paramount importance. Standardization ensures consistency and accuracy in the information provided to HCPs [30]. By establishing standardized protocols and materials for educational interventions, HCPs can rely on consistent and reliable information when making decisions about vaccinations. This consistency helps to avoid confusion or misinformation among healthcare professionals, leading to more informed decision-making. It will therefore be important to make these interventions not just part of an isolated project but periodic and constitutive of medical training. Although this study provides valuable insights into the safety and adherence of the intranasal spray influenza vaccine (qLAIV) among the paediatric population, there are several limitations that should be acknowledged. First, despite the study was conducted in the Province of Palermo, representing one quarter of the entire Sicilian population, it may not be representative of other regions or countries with different healthcare systems, demographics, and cultural factors. Therefore, the findings may not be generalizable to broader populations. The study lacks also a control group for comparison, which makes it challenging to attribute changes in vaccine adherence or safety outcomes solely to the intervention. Moreover, the study could denote a possible selection bias, because it was not possible to extensively control for potential confounding variables, such as socioeconomic status, parental education level, or access to healthcare services, which could have influenced vaccine adherence and safety outcomes.

## Conclusion

This study successfully bolstered paediatric influenza vaccination rates for children aged 2 to 14, particularly within the urban and provincial areas of Palermo. Notably, more than 80% of the participants opted for the paediatric intranasal spray vaccine, indicating a parental inclination towards this formulation over QIVsd. The occurrence of minimal adverse events, all of which were non-serious, served as compelling evidence for the vaccine’s high safety profileThe training sessions conducted as part of our project played a pivotal role in ensuring that paediatricians remained abreast of the latest information on vaccine safety and importance. This equipped them with the necessary tools to furnish parents with comprehensive information, thereby facilitating secure and informed decision-making regarding vaccination in their practices.

Finally, the present project confirmed that the qLAIV could play a key role in increasing influenza vaccination coverage in the not immunocompromised paediatric population aged older than 24 months of age, due to high rate of acceptance among children and parents and limited rate of adverse events.

## Data Availability

Data obtained in the present study are available upon request to the corresponding author.
